# Structural and biochemical investigations of a HEAT-repeat protein involved in the cytosolic iron-sulfur cluster assembly pathway

**DOI:** 10.1038/s42003-023-05579-3

**Published:** 2023-12-18

**Authors:** Sheena Vasquez, Melissa D. Marquez, Edward J. Brignole, Amanda Vo, Sunnie Kong, Christopher Park, Deborah L. Perlstein, Catherine L. Drennan

**Affiliations:** 1 Department of Biology, Massachusetts Institute of Technology, Cambridge, MA 02139 USA; 2 Department of Chemistry, Boston University, Boston, MA 02215 USA; 3 MIT.nano, Massachusetts Institute of Technology, Cambridge, MA 02139 USA; 4 Howard Hughes Medical Institute, Massachusetts Institute of Technology, Cambridge, MA 02139 USA; 5 Department of Chemistry, Massachusetts Institute of Technology, Cambridge, MA 02139 USA

**Keywords:** Metalloproteins, Cryoelectron microscopy

## Abstract

Iron-sulfur clusters are essential for life and defects in their biosynthesis lead to human diseases. The mechanism of cluster assembly and delivery to cytosolic and nuclear client proteins via the cytosolic iron-sulfur cluster assembly (CIA) pathway is not well understood. Here we report cryo-EM structures of the HEAT-repeat protein Met18 from *Saccharomyces cerevisiae*, a key component of the CIA targeting complex (CTC) that identifies cytosolic and nuclear client proteins and delivers a mature iron-sulfur cluster. We find that in the absence of other CTC proteins, Met18 adopts tetrameric and hexameric states. Using mass photometry and negative stain EM, we show that upon the addition of Cia2, these higher order oligomeric states of Met18 disassemble. We also use pulldown assays to identify residues of critical importance for Cia2 binding and recognition of the Leu1 client, many of which are buried when Met18 oligomerizes. Our structures show conformations of Met18 that have not been previously observed in any Met18 homolog, lending support to the idea that a highly flexible Met18 may be key to how the CTC is able to deliver iron-sulfur clusters to client proteins of various sizes and shapes, i.e. Met18 conforms to the dimensions needed.

## Abstract (web summary)

Structural and biochemical studies of Met18, a CIA Pathway heat repeat protein, show that Met18 forms different oligomers, has a flexible N-terminus that binds client protein Leu1, and a C-terminus that binds CIA protein Cia2.

## Introduction

**Fig. 1:**
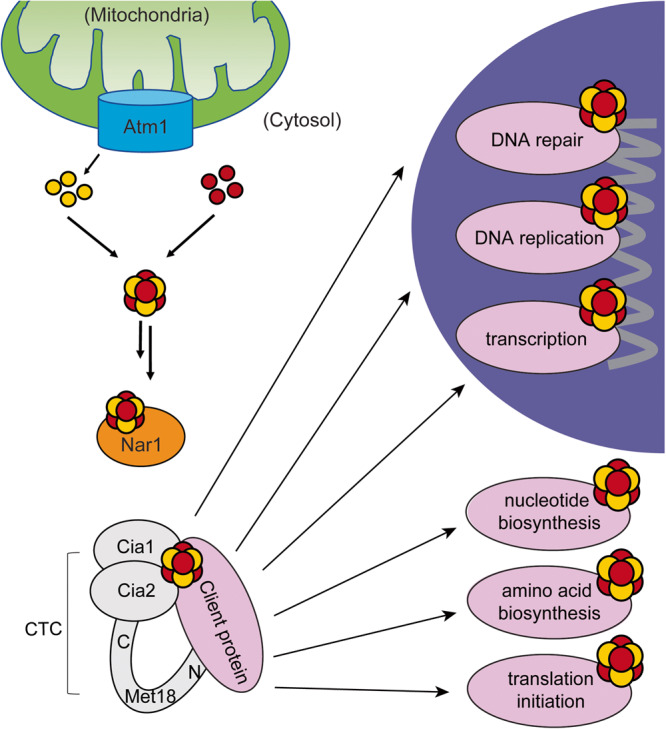
The CIA Pathway Targeting Complex (CTC) delivers iron-sulfur clusters to clients in the cytosol and nucleus. A sulfur-containing metabolite is exported from the mitochondria through Atm1 and combined with iron to assemble into an iron-sulfur cluster via upstream factors in the CIA pathway. The CTC consisting of Met18-Cia2-Cia1 is believed to receive the mature iron-sulfur cluster from Nar1 and deliver this cluster to apo-clients required for a variety of biochemical processes. Red and yellow circles depict iron and sulfur, respectively. Model for CTC complex is based on the 12-Å cryo-EM map for the apo CTC-primase complex^5^.

Iron-sulfur (Fe-S) clusters are versatile and important cofactors that allow numerous proteins to carry out their functions^1–3^. Fe-S clusters are involved in electron transport, gene expression regulation, and enzyme catalysis^1,2^. Although the importance of mitochondrial Fe-S proteins for primary metabolism is well-known, the prevalence of extramitochondrial Fe-S enzymes has been underappreciated. Cytosolic and nuclear Fe-S proteins are essential for life-sustaining processes such as: DNA replication and repair, transcription, translation, and nucleotide and amino acid biosynthesis [1-3]. Examples include DNA and RNA polymerases; primases; some helicases, such as the Rad3 component of the TFIIH complex required for transcription and nucleotide excision repair; Rli1, which is important for export of ribosomal subunits from the nucleus and promotion of translation initiation complex assembly; and Leu1, an isopropylmalate isomerase that catalyzes the second step in the leucine biosynthetic pathway^1,2,4,5^. These cytosolic and nuclear Fe-S cluster-containing proteins obtain their cofactors via the cytosolic iron-sulfur cluster assembly (CIA) pathway^1–3^ (Fig. 1). Due to the importance of the CIA pathway for maturation of essential Fe-S enzymes, CIA factors are highly conserved from yeast to humans. In the last step of the pathway, the CIA targeting complex (CTC) identifies apo-client proteins and delivers a mature iron-sulfur cluster^1–3^. The *Saccharomyces cerevisiae* CTC is comprised of the proteins Met18 (MMS19 in humans), Cia1 (CIAO1 in humans) and Cia2 (CIAO2B/CIA2B in humans). It is not well understood how the CTC recognizes its clients, now numbering > 30 eukaryotic proteins. A molecular level understanding of how the CTC controls flux through the CIA pathways is critical for elucidating how dysregulation of CIA impacts chromosomal integrity and contributes to carcinogenesis^4,6–8^.

Here, we focus on the CTC protein Met18 from *S. cerevisiae* (ScMet18), which was first discovered as a methionine auxotrophic^9^ and methyl methanesulfonate-sensitive (MMS) mutant^10^, and later shown to be involved in recognition of apo-client proteins in the cytosol and nucleus^4–6^. Met18 is a 118 kDa alpha solenoid protein comprised entirely of HEAT (Huntingtin, elongation factor 3 (EF3) repeats, protein phosphatase 1A, and the yeast kinase TOR1) repeats. Arrays of HEAT-repeats, each comprising two alpha helices, are well known to mediate protein-protein interactions and are often a part of large protein complexes^11^, consistent with Met18’s proposed role in client identification during the final step of the CIA pathway. Structural data on Met18 and on the CTC complex are limited, but recent structural work from Kassube and Thomä^5^ has provided key insights. These authors were able to determine a 3.6 Å resolution crystal structure of a complex between Cia2b-Cia1 from *Drosophila melanogaster* (DmCia2b-DmCia1) and Met18 from *Mus musculus* (MmMet18)^5^. This hybrid-species structure was a dimer of CTC units, mediated by homodimeric interactions between the two Met18 protomers as well as the two Cia2b proteins. Each CTC unit showed Met18 bound to Cia2b via Met18’s final HEAT-repeat (helices 45 and 46), Cia2b bound to Cia1, with no direct interactions between Met18 and Cia1^5^, in agreement with previous biochemical studies^12–14^. Notably, lysine residues from helices 45 and 46 of *Homo sapiens* Met18 (K993, K1002, K1007, K1008, and K1013, numbering from human Met18) which were demonstrated to be important for the HsMet18-HsCia2b interaction via co-immunoprecipitation studies, are the target of ubiquitination^15^, suggesting that CTC complex formation may protect Met18 from degradation in eukaryotes; three (K1008, K1009, and K1014, numbering from *S. cerevisiae* Met18) of the five lysines are conserved between yeast and humans (Fig. S1).

To further examine how CTC interacts with its clients, Kassube and Thomä obtained low-resolution (8-12 Å) cryo-EM data on human CTC in the presence of client proteins DNA2 (CTC-DNA2) and the PriS-PriL primase heterodimer (CTC-primase)^5^. The arrangement of MmMet18-DmCia2b-DmCia1 observed in the hybrid-species crystal structure appeared to be conserved in the human CTC, however, the CTC dimer observed with the mouse and fly orthologs is not observed in the client-bound state. The primase heterodimer could be modeled into the cryo-EM maps, but DNA2 could not. Despite this limitation, these client-bound CTC maps supported the proposal that clients dock on the CTC via a bipartate interaction using the N-terminus of Met18 together with the conserved patch on the side of Cia1^5,14^. Since Met18 was found to have a different superhelical curvature in all CTC structures, Kassube and Thomä proposed that this flexibility could allow for Met18 to recognize a variety of client proteins. Despite these important insights into the function of the CTC, the CTC-primase structure was not in a relevant state for Fe-S cluster transfer: no cluster was bound; and the primase [4Fe-4S] cluster binding site was 70 Å from the proposed cluster binding site on Cia2b^12,16^. Thus, a conformational change or additional factors are needed for Fe-S cluster transfer from the CTC to client proteins. The observed flexibility of Met18 in the different CTC structures^5^ suggested Met18 could flex to bring Cia2 closer to the client to afford Fe-S cluster transfer.

In this study, we use a combination of cryo-EM, negative stain EM, pulldown assays, and mass photometry to biophysically investigate *S. cerevisiae* Met18 (ScMet18). To interrogate the proposal that flexibility is key to Met18’s function^5^, we used two methods of cryo-grid preparation to determine structures of ScMet18 in isolation. We find that ScMet18 adopts a different conformation from those previously observed for the human and mouse homologs that allows it to form hexamers and tetramers in the absence of *S. cerevisiae* Cia2 (ScCia2). The addition of ScCia2 causes these higher order oligomeric states of ScMet18 to dissociate to form Met18-Cia2 complexes. Through pull-down assays, we identify residues at ScMet18’s N-terminus that are important for binding client protein *S. cerevisiae* Leu1 (ScLeu1) and at Met18’s C-terminus that are required to bind ScCia2. We compare these structures to the previously determined structures of Met18 in complex with other proteins^5^. Insights from these comparisons and from the biochemical data into Met18 regulation and function are described herein.

## Results

### 3.3 Å resolution cryo-EM structure of ScMet18 in a hexameric state was obtained using Vitrobot plunged grids

To gain insight into the structure of ScMet18, a member of the CTC in the CIA pathway (Fig. 1), we purified recombinantly expressed SUMO-tagged ScMet18, cleaved the SUMO tag, and used untagged ScMet18 to prepare cryo-EM grids with a Vitrobot cryoplunger. Due to preferential orientation of ScMet18 particles on grids prepared using the Vitrobot, we collected 3 datasets on a Titan Krios (totaling 8031 micrographs) applying 0°, 25°, and 40° stage tilts to obtain additional particle orientations (Fig. S2 and Table 1). We processed the tilted data separately and obtained 2D class averages of particles. We then pooled the particles from 2D class averaging for 3D ab initio modeling. We first applied C1 symmetry and observed 2-fold and 3-fold symmetry in the map (Fig. S2). Therefore, D3 symmetry was applied throughout the rest of the data processing to generate Map 1 (Fig. S2). Map 1 is at 3.3 Å resolution (Fig S3a and Table 1). Due to air-water denaturation of particles and/or flexibility of the N-terminus, we applied masked focus classification to Map 1 during data processing (Fig. S2). Three classes were generated, and the particles and map from the class with the best density at the N-terminus (Class 3) was used for masked refinement to generate Map 2 (Fig. S2). Map 2 is at 3.6 Å resolution (Fig S3b and Table 1). We combined Map 1 and Map 2 to create a map for model building using WARP Frankenamp^17^ (Fig. S2), and used this combined map, along with Maps 1 and 2, to manually build residues 138–1029. The MmMet18 structure (PDB 6TC0) was docked into the combined map and used as a starting model for building residues 9–137 (Table 1).

**Table 1:** Collection and processing of data from both Vitrobot (map 1 and 2) and chameleon-prepared (map 3 and 4) grids of ScMet18.

Image parameters & 3D reconstruction				
	0°	25°	40°	chameleon
Microscope	FEI Titan Krios	FEI Titan Krios	FEI Titan Krios	Talos Arctica G2
Camera	Gatan K2	Gatan K2	Gatan K2	Falcon 3EC
Acceleration voltage (kV)	300	300	300	200
Pixel size (Å)	1.059	1.059	1.059	1.5998
Energy filter slit width	n/a	n/a	n/a	n/a
Magnification	130000x	130000x	130000x	92000x
Defocus range (μm)	−0.7 to −2.5	−0.8 to −2.4	−0.8 to −2.4	−1.3 to −3.4
Number of frames	45	45	45	14
Exposure time (s)	9.00	7.65	7.65	7.00
Total exposure (e-/Å)	49.59	49.68	49.68	53.26
Total micrographs collected	3968	1783	2280	483
Automation software	SerialEM	SerialEM	SerialEM	EPU
	Map 1	Map 2	Map 3	Map 4
Particles In final 3D reconstruction	171255	44707	36582	30104
Symmetry imposed	D3	D3	D3	C1
Map sharpening B-factor	−104	−66		
Estimated accuracy of rotations (degrees)	0.49	0.45	2.1	3.43
Estimated accuracy of translations (pix)	0.23	0.23	0.94	1.88
Unmasked resolution at 0.5/0.143 FSC (Å)	4.17/3.70	5.56/4.17	10/9.1	15/13
Masked resolution at 0.5/0.143 FSC (Å)	5.26/3.3	6.67/3.6	9/8.4	13/12
Local resolution range (Å)	3.21 - 6.45	3.43 - 7.46	0 - 16.42	11–15.47
Microscope used	FEI Titan Krios	FEI Titan Krios	Talos Arctica	Talos Arctica
Oligomeric state	hexamer	hexamer	hexamer	tetramer
EMDB accession code	EMD-42512	EMD-42513	EMD-42514	EMD-42511

**Fig. 2:**
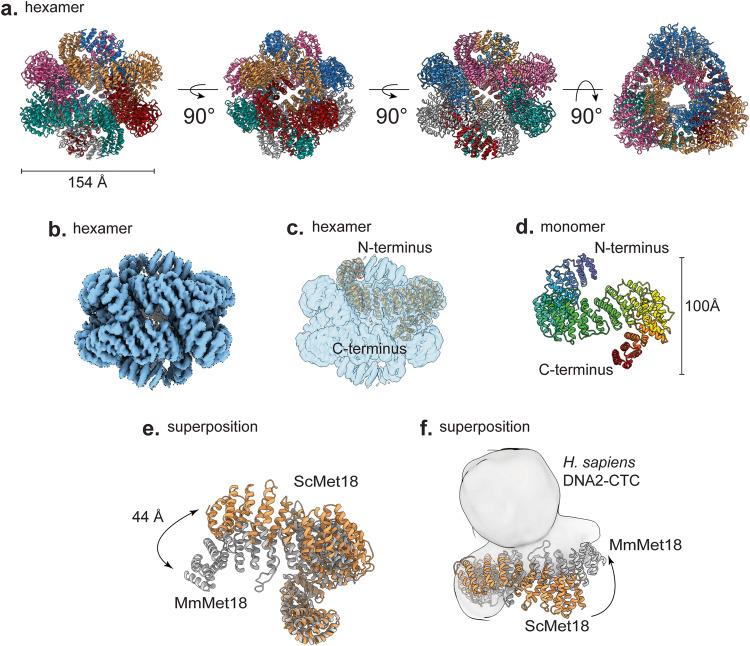
ScMet18 forms hexamers and has a flexible N-terminus. **a** Views of the 2-fold and 3-fold axes of the hexameric form of ScMet18 with each chain colored differently. **b** Frankenmap combined cryo-EM Map 1 and Map 2 of the Vitrobot-plunged cryo-EM grids of ScMet18**. c** Chain A of the hexamer is shown in sandy brown in the Frankenmap combined map (transparent blue) without any other chains for clarity. The N-terminus is exposed, and the C-terminus is buried. **d** Protomer of the hexamer shown in rainbow in the same orientation as in **c**. **e** The curvature of Met18 is different in the ScMet18 hexamer compared to the MmMet18 structure in complex with Cia2-Cia1. If aligned by the C-termini, the N-termini of ScMet18 and MmMet18 are 44 angstroms apart. **f** ScMet18 and MmMet18 are aligned by their C-termini and shown in the CTC-DNA2 complex cryo-EM density^5^. Neither structure is a good fit, indicating that additional conformational rearrangement of Met18 must occur.

To the best of our knowledge, our cryo-EM structure shows for the first time that ScMet18 can form an intertwined hexamer in the absence of other proteins (Fig. 2a and Supplemental Movie 1). Our final model contains six copies of Met18 with each protomer consisting of residues 9-1029 with two loops missing at residues 225–241 and 315–337 (Fig. 2 and Table 2). The hexamer is 154 Å long and has both 3-fold and 2-fold symmetry (Fig. 2a, b). For all chains, the N-terminus is solvent-exposed, and the C-terminus is buried (Fig. 2c). Each chain of the hexamer interacts with 4 other chains (i.e., chain A touches chains B, C, D, and F, but not chain E (Fig. S4a)). The hexamer is comprised of trimers and dimers where chains A, B, and C and chains D, E, and F can form trimers, and chains A and F, B and E, and C and D form dimers (Fig. S4a). Notably, the dimer from ScMet18 hexamer is not the same as the dimer observed in the crystal structure of MmMet18 in complex with DmCia2b-DmCia1(Fig. S4a, b). Numerous contacts are made between the ScMet18 dimers (Fig. S5). Aliphatic residues pack against each other at the center of the dimer interface, and this hydrophobic patch is flanked by salt bridges (Fig. S5).

**Table 2:** Cryo-EM data collection, refinement and validation statistics.

	#1 Met18 hexamer (EMDB-42510) (PDB 8USP)	#2 Met18 tetramer (EMDB-42511) (PDB 8USQ)
Data collection and processing		
Magnification	130,000x	92,000x
Voltage (kV)	300	200
Electron exposure (e–/Å^2^)	49.59/49.68	53.26
Defocus range (μm)	−0.7–−2.5/−0.8–−2.4	−1.2–−3.3
Pixel size (Å)	1.059	1.5998
Symmetry imposed	D3	C1
Initial particle images (no.)	379,779	87,417
Final particle images (no.)	171,255	30,104
Map resolution (Å)	3.3	12.8
FSC threshold	0.143	0.143
Map resolution range (Å)	3.21–6.45	11–15.47
Refinement		
Initial model used (PDB code)	6TC0	6TC0
Model resolution (Å)	3.3	12.8
FSC threshold	0.143	0.143
Model resolution range (Å)	3.21–6.45	11–15.47
Map sharpening *B* factor (Å^2^)	-104	
Model composition		
Non-hydrogen atoms	7896	7901
Protein residues	982	982
Ligands	0	
* B* factors (Å^2^)		
Protein	222.61	703.77
Ligand	NA	
R.m.s. deviations		
Bond lengths (Å)	0.002	0.003
Bond angles (°)	0.598	0.754
Validation		
MolProbity score	1.58	2.29
Clashscore	7.48	23.40
Poor rotamers (%)	0.02	0.00
Ramachandran plot		
Favored (%)	97.03	93.44
Allowed (%)	2.94	6.56
Disallowed (%)	0.03	0.00

ScMet18 is similar by sequence (25% identity) and by structure to MmMet18^5^ (Figs. 2e and S1). ScMet18 has a curved S-shaped structure comprised of 23 HEAT-repeat (HR) units; each HR contains two α-helices that are connected by loops (Figs. 2d and S4c). In total, there are 50 helices in ScMet18: four 3_10_ helices and 46 α-helices in ScMet18. MmMet18 also has 23 HR units (Figs. 2e and S4c) and 46 α-helices. The S-shape solenoid of ScMet18 forms a left-handed superhelical structure with a length of 110-112 Å across (Fig. 2d). The curvature of ScMet18 differs from what has been observed previously with the MmMet18 protein in complex with DmCia2-DmCia1 (Fig. 2e and 2f); there is a 44 Å shift in the N-terminus of ScMet18 compared to MmMet18 (Fig. 2e).

### ScMet18 tetrameric state is more apparent in grids prepared using the chameleon

To address the preferred orientation issues that we observed with the Vitrobot-prepared cryo-EM grids (Fig. S6a), we prepared grids of ScMet18 (SUMO-tag was cleaved) using a chameleon, a commercially available piezoelectric cryo-EM plunger that is based on the SpotitOn plunger developed by Carragher and co-workers^18^. We collected a dataset on a Talos Arctica (totaling 463 micrographs) and processed this dataset applying D3 and C1 symmetry to generate Maps 3 and 4, respectively. Map 3, a map showing the hexameric state of ScMet18, is at 8.46 Å resolution (Figs. S7–S8). This map compares well to Maps 1 and 2 showing 2-fold and 3-fold symmetry. The lower resolution can be attributed to the lower number of particles and higher pixel size used during data collection and processing. Despite the different plunging method used, preferred orientation was still an issue, albeit much less of an issue **(**Fig. S6b).

To our surprise, the data collected from the chameleon sample had tetramers present in addition to the hexamers observed in the Vitrobot dataset. The tetramer contains 2-fold symmetry and was refined to 12.77 Å (Figs. S7–9 and Table 2). Both the hexamer and the tetramer have similar overall dimensions and their chains have similar curvatures (Figs. 2a and S9), despite the fact that the tetramer contains two fewer chains. As a result, the tetramer is less tightly packed than the hexamer, especially around the more exposed N-termini (Fig. S9), since its protomers are shifted away from one another as compared to their relative orientation in the hexamer (Figs. 2a and S9).

With the discovery that ScMet18 can form a tetramer, we went back to re-analyze the Vitrobot-prepared samples, suspecting that tetrameric states were also present, but that there were not enough intact tetramers for robust class averages to be generated. Since we previously observed that chameleon-prepared samples suffer less from particle denaturation than those prepared on a Vitrobot^19^, we speculate that our ability to readily visualize the tetrameric state in the chameleon-prepared samples is due to less particle denaturation. The protomers of the tetrameric state appear to be less tightly packed (Fig. S9) than protomers of ScMet18 in the hexameric state, and thus the ScMet18 tetrameric state may be more prone to issues of particle denaturation. Short-lived and/or thermodynamically unstable states can provide valuable insight into protein assembly/disassembly processes but are hard to capture experimentally. The chameleon may prove beneficial in allowing for the visualization of such structural snapshots.

### Pulldown assays suggest that conserved residues at the N-terminus of ScMet18 are involved in client protein binding

Previous CTC structure determinations and biochemical studies indicated that the conserved N-termini of MmMet18 and HsMet18 (HEAT-repeat (HR)^2–8^; Interpro domain IPR029240, Fig. S4c) are associated with client protein binding, and the final HR of Met18 is associated with Cia2 binding^5^. However, co-immunoprecipitation studies using truncated Met18 variants and various clients have generated conflicting information about which HR is involved in binding various clients^14,20^. Furthermore, Met18 also contains a second conserved domain comprising HR14-22 (MMS19, C-terminal domain; IPR024687, Fig. S4c). Currently, the function of this region of Met18 is not known; however, most of the interchain hydrogen bonding interactions in the ScMet18 hexamer involve these conserved regions, whereas the poorly conserved regions, HR1 and HR9-13, contribute little to oligomer formation (Fig. S4c). These data support the proposal that these conserved regions of Met18 are most important for their ability to mediate protein-protein interactions between the CTC and clients.

**Fig. 3:**
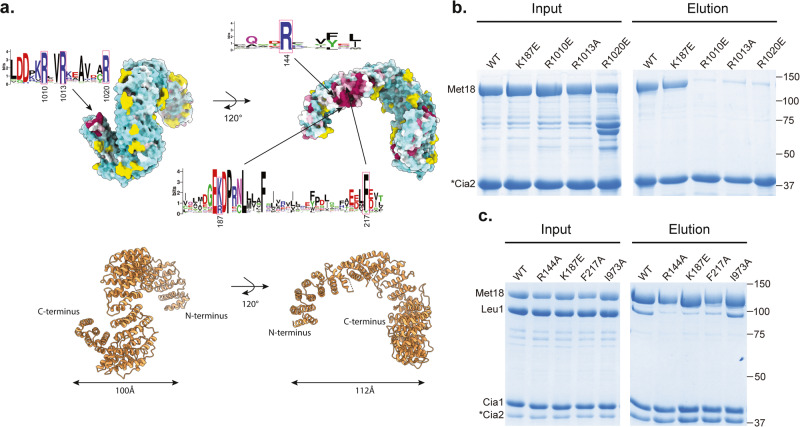
Conserved residues at the N-terminus of ScMet18 and at the C-terminus are involved in client protein binding and Cia2 binding, respectively. **a** Structure of protomer colored by conservation (top) with ribbon drawing (bottom). At the N-terminus, the most conserved residues include R144 and G185-F187. At the C-terminus, the most conserved residues include L1005-R1020. Conservation is colored from most conserved (magenta) to least conserved (light blue) in the maps. Conservation correlates to the size of the letters in the sequence logo (i.e., largest letters are most conserved). Boxed letters with residue numbers indicate the residues that were mutated in this study. Sequence logo was made using WebLogo (https://weblogo.berkeley.edu/logo.cgi). **b** ScMet18-ScCia2 interaction analysis. The indicated ScMet18 variant was mixed with Strep-tagged ScCia2 bait (*) and chromatographed through Streptactin resin. The Streptactin column input (left panel) and elution (right panel) fractions were analyzed by SDS-PAGE. **c** ScLeu1 interaction with the CTC. Experiments were performed as described in **b** except ScLeu1 and ScCia1 were included in addition to the indicated ScMet18 variant and the ScCia2 bait. Unedited images of **b**, **c** are shown in Fig. S10.

To test the importance of residues in the N-terminal (HR4-6) and C-terminal (HR22-23) regions of ScMet18 for facilitating protein-protein interactions, we made the following residue substitutions: R144A in HR4; K187E in HR5; F217A in HR6; I973A in HR22; and R1010E, R1013A, and R1020E in HR23 and used pulldown assays to investigate ScMet18’s ability to bind the CTC subunit ScCia2 and the client protein ScLeu1, previously shown to bind to ScMet18 in the presence of both ScCia1 and ScCia2^13^ (Figs. S4c and 3). Since ScCia2 and ScMet18 form a stable binary complex^13^, we began these studies by monitoring the ability of a strep-tagged ScCia2 bait to pulldown ScMet18, which also has an N-terminal His-SUMO tag to aid with heterologous protein expression and solubility. The C-terminal ScMet18 variants R1010E, R1013A, and R1020E did not pulldown with ScCia2 to a substantial extent, whereas the variants R144A, K187E, F217A, and I973A were able to be pulled down with ScCia2 in amounts comparable to that of wild type ScMet18 (Fig. 3b, c). To evaluate the interaction between ScMet18 (SUMO-tagged) and the ScLeu1 client, the ScCia1 and ScCia2 proteins were included because the full CTC is required to pull down Leu1. Thus, only the ScMet18 variants competent to bind ScCia2 bait could be tested for their ability to pull down the CIA client ScLeu1. Each of the N-terminal variants, R144A, K187E, and F217E, were defective in ScLeu1 binding as compared to wild-type ScMet18 and a C-terminal variant, I973A (Fig. 3c). These results are consistent with the proposal^5^ that the N-terminus of Met18 is involved in client protein binding and that the C-terminus contacts Cia2.

**Fig. 4:**
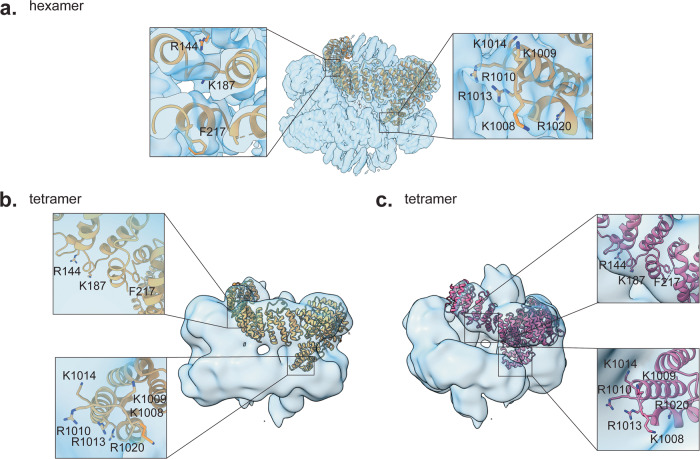
Location of conserved residues in the hexameric and tetrameric states of ScMet18. **a** Hexamerization of ScMet18 appears to limit access to conserved N-terminal residues R144, K187, and F217 and block access to conserved C-terminal residues K1008, K1009, R1010, R1013, K1014, and R1020. One protomer is shown in sandy brown in the map (transparent blue). **b**, **c** Tetramerization of ScMet18 also limits access to conserved N-terminal residues R144, K187, and F217 and blocks access to conserved C-terminal residues K1008, K1009, R1010, R1013, and K1014 in the sandy brown protomer, and blocks access to conserved C-terminus residues K1008, K1009, R1010, R1013, K1014, and R1020 in the hot pink protomer. Conserved N-terminal residues R144, K187, and F217 of the hot pink protomer are more accessible than they are in any of the hexamer protomers.

Notably, residues that appeared to be involved in binding Cia2 (R1010, R1013, R1020) are buried in both the hexameric (Fig. 4a) and tetrameric states (Fig. 4b, c) of ScMet18, indicating that ScCia2 cannot bind to ScMet18 without disrupting these higher order oligomeric states. Similarly, the N-terminal residues whose substitution was shown above to impair association with client protein ScLeu1 (R144, K187, F217) are partially buried in the hexamer and in two of the chains of the tetramer (Fig. 4). In the other two chains of the tetramer, these N-terminal residues are mostly exposed (Fig. 4c). Thus, we propose that the higher order oligomeric states of ScMet18 must disassemble to allow for the formation of a high-affinity active CTC complex.

### The addition of ScCia2 disassembles the higher order oligomeric states of ScMet18

**Fig. 5:**
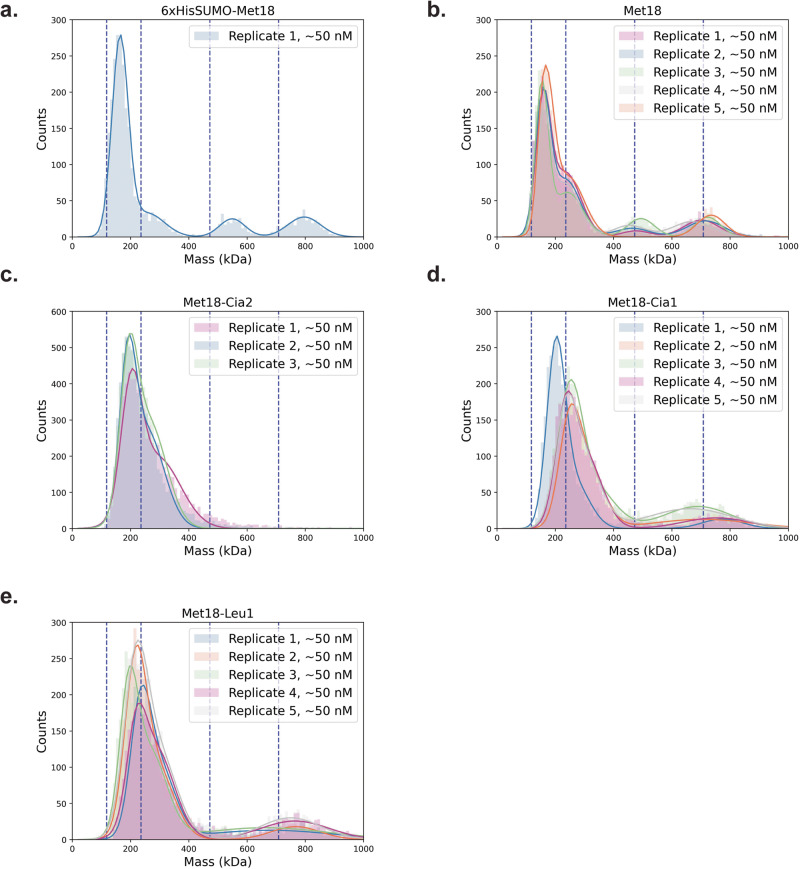
Mass photometry of ScMet18 alone and in the presence of ScCia2, ScCia1, and ScLeu1. **a** Control: presence of SUMO-tag does not change the oligomeric distribution of ScMet18. Like untagged ScMet18 (see panel **b**), SUMO-tagged ScMet18 is a mixed of monomers, dimers, tetramers, and hexamers. The molecular weights (MWs) calculated by MP for SUMO-tagged ScMet18 are 164 kDa, 256 kDa, 549 kDa, and 796 kDa, which agree well with the predicted MWs of the monomer (131 kDa), dimer (262 kDa), tetramer (524 kDa), and hexamer (786 kDa) of the SUMO-tagged ScMet18. Dotted lines correspond to ScMet18 monomer, dimer, tetramer and hexamer (left to right). **b** Untagged ScMet18 is a mixture of monomers, dimers, tetramers, and hexamers. The MWs observed are 157 ± 3 kDa, 236 ± 4 kDa, 472 ± 17 kDa, and 719 ± 14 kDa for untagged ScMet18, which corresponds to the calculated MWs of untagged ScMet18 monomer (118 kDa), dimer (236 kDa), tetramer (472 kDa), and hexamer (708 kDa), respectively. Five replicates are shown with each replicate in a different color. **c** Untagged ScMet18 in the presence of ScCia2 shows a loss of higher order oligomeric species. Experimental MWs are 195.4 ± 6 kDa and 269.3 ± 20 kDa. Three replicates are shown. **d** Untagged ScMet18 in the presence of ScCia1 retains higher order oligomers compared to ScMet18 in presence of ScCia2 (panel **c**). Five replicates are shown. **e** Untagged ScMet18 in the presence of ScLeu1 retains higher order oligomers compared to ScMet18 with ScCia2 (panel **c**). Five replicates are shown.

To investigate which, if any, CTC proteins facilitate disassembly of the ScMet18 tetramer or hexamer en route to an active CTC complex, we turned to mass photometry (MP). Although higher order oligomeric states of ScMet18 dominate our cryo-EM data, we find that when ScMet18 is in solution, it is present as a mixture of monomers, dimers, tetramers, and hexamers. This mixture exists regardless of whether or not the SUMO tag is present (Fig. 5a, b). However, when ScCia2 is titrated into the solution of untagged ScMet18, higher order forms of ScMet18 disappear (Fig. 5c). Interestingly, ScCia1 and ScLeu1 do not show as dramatic of an effect on the higher order states of ScMet18. Whereas the hexameric state of ScMet18 remains apparent, the tetrameric state of ScMet18 does not appear to be present (Fig. 5d, e). It is perhaps not surprising that ScCia1 has less of an impact on ScMet18’s oligomeric state than ScCia2, given that Cia1 does not bind directly to Met18^5,13^. It is more surprising that ScLeu1 does not appear to substantially change the oligomeric state of ScMet18 given that our data indicate direct binding. That being said, the binding studies with ScLeu1 and ScMet18 were performed in the presence of both ScCia1 and ScCia2, and it has been shown that high-affinity complex formation between CTC proteins and a client protein requires Cia1, Cia2, and Met18^5,13^. Collectively, these data indicate that ScCia2, which serves to disassemble the ScMet18 hexamer, plays a role in preparing Met18 for high-affinity client binding.

## Discussion

Metallocofactor delivery can be highly specialized, with one protein delivering one cofactor to one client protein, or delivery can be less specialized. CTC proteins standout in that they deliver Fe-S clusters to more than 30 clients^1,2^. CTC client proteins perform divergent functions including amino acid and nucleotide biosynthesis, ribosome biogenesis, and DNA replication and repair. These clients are structurally distinct, raising the questions: how do the same three CTC proteins, Met18, Cia1, and Cia2, recognize clients of different sizes and shapes while avoiding holo-clients and nonclients? Also, what happens to the CTC in between ‘jobs;’ are CTC proteins protected from degradation so that they are ready for the next client, and if so, how does the CTC reassemble in the presence of a new client? Here, we have used a variety of biophysical methods to probe these questions and better understand the properties of Met18 that support its function.

Our work, along with previous studies^5^, provides insight into the question of how the CTC recognizes and accommodates vastly different client proteins. Previous studies have indicated that each CTC subunit (Met18, Cia1, Cia2) contributes to client identification, leading to a model that two or more, low affinity, relatively promiscuous interactions are leveraged by the CTC to bind its clients with high affinity and specificity^5,14,20–22^. Indeed, Kassube and Thomä demonstrated that high-affinity client binding is cooperative and requires the full CTC complex^5^. We have found previously that the full CTC leads to higher affinity Leu1 binding^13^, and we show here that residues in HEAT-repeats 4-6 of Met18’s conserved N-terminal region are required for that Leu1 binding. The residues, R144, K187, and F217, are found in the center of a large patch of conserved residues. Interestingly, this conserved patch is adjacent to the putative client binding sites predicted by low-resolution cryo-EM data on client-CTC complexes^5^, further corroborating the role of this region of Met18 in client protein recognition. With respect to the binding of a wide variety of client proteins, our studies highlight Met18’s flexibility and show that Met18’s curvature adapts to its binding partners, supporting the proposal that Met18’s conformational flexibility is key for the recognition of clients of various sizes and shapes. This role is not a new one for a HEAT-repeat protein. Karyopherins, the HEAT-repeat proteins involved in transporting molecules between the nucleus and cytoplasm, rely on flexibility for the binding and release of cargo during nuclear transport^11^.

The hexameric and tetrameric forms of Met18 observed in our cryo-EM studies cannot be the forms of Met18 to which client proteins bind given that oligomerization partially buries R144, K187, and F217. Instead, we suggest that oligomerization could protect Met18 from degradation when the CTC is paused between clients and/or protect from interactions with nonclients or holo-clients. When a maturase has one job (deliver adenosylcobalamin to its client mutase)^23^, job completion and maturase degradation could go hand-in-hand, but for a maturase system with multiple clients, there is a logic to keeping the maturase available for a new client but not so available that nonclient or holo-protein interactions occur. Previous studies have demonstrated that degradation of the CTC subunits, particularly Met18 and Cia2, is regulated in part by CTC complex formation^5,14,15^. When the CTC is ‘working’, the close juxtaposition of Met18’s C-terminal ubiquitination sites and its Cia2 binding site is thought to prevent ubiquitination^5^. Our work provides a possible extension to this model; when the CTC is ‘paused’ between clients, the burial of Met18’s C-termini within a higher order oligomer should limit ubiquitination and allow Met18 to wait for the next client. The proposal that protein oligomerization/protein complex formation can provide protection from degradation is not new. For example, hexamerization of proinsulin shields it from protease digestion^24^. When released into the blood, the hexamer dissociates into active monomers that can be degraded^24^. Also, complex formation between WRB (tryptophan-rich basic protein) and CAML (calcium-modulating cyclophilin ligand), which together serve as an insertase for tail-anchored proteins, imparts greater stability^25^. When the WRB-CAML complex is unassembled, each subunit is differentially recognized and degraded^25^.

**Fig. 6:**
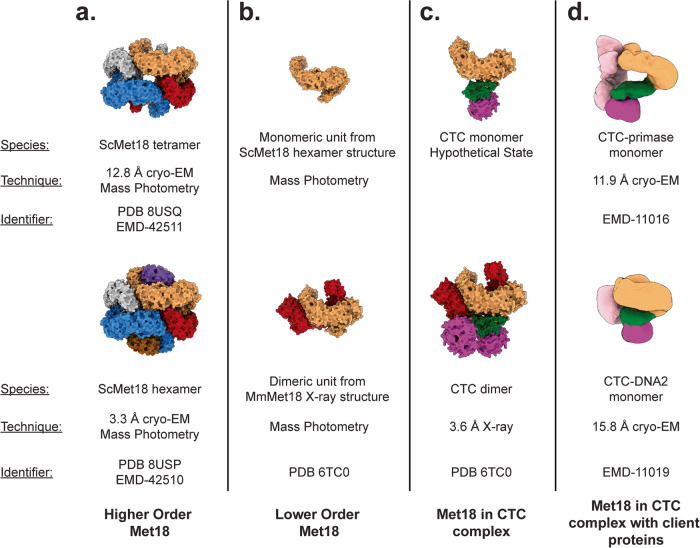
Observed (and one hypothetical) states of Met18. **a**–**d** The sandy brown Met18 protomer is shown in roughly the same orientation to facilitate comparison of the different observed structures. Other Met18 protomers are colored red-sox red, dodger blue, brown, purple, dark brown, and silver. Cia1 is magenta, Cia2 is green, and client proteins (primase and DNA2) are light pink.

After decades with little to no structural data on CTC proteins, the glass ceiling has cracked. Structural snapshots (Fig. 6) are becoming available and key biochemical analyses are accompanying those snapshots. However, there is still much to do. For example, studies assessing the role of Met18 hexamerization/tetramerization (Fig. 6a) in protection of Met18 from ubiquitination and protein degradation will test hypotheses presented here. Although this work indicates that Cia2 shifts the Met18 oligomeric state equilibrium away from the higher order states toward ‘active’ states, molecular details for how Cia2 accomplishes this feat are lacking. Also, we have no structures of Met18 in a monomeric or dimeric state in the absence of Cia1 and Cia2, although we know these oligomeric states exist (Fig. 6b). Further, we do not know if client proteins bind to a monomeric or dimeric CTC (Fig. 6c). A crystal structure provides a view of a dimeric CTC (Fig. 6c), whereas EM studies indicate that a monomeric CTC may bind to client proteins (Fig. 6c)^5^, leaving the question of oligomeric state relevance open. Importantly, the CTC-primase structure shown in Fig. 6d cannot be the final state. As noted in reference^5^, the CTC-primase structure is not a competent state for Fe-S cluster transfer, given that the site to which the Fe-S cluster binds on the primase is 70-Å from the cysteine on Cia2 that is a proposed cluster ligand. It is likely that multiple states of a CTC-client complex exist: a state to which an Fe-S cluster is delivered, presumably by Nar1 (Fig. 1); a holoCTC-client protein state in which the cluster is bound to Cia2 or Cia2-Cia1; and another holoCTC-client protein state in which the cluster is bound to the client. Thus, although we now have a wealth of structural data compared to a few years ago, there are many more snapshots to be obtained in order for us to understand the fundamental, yet enigmatic, biological process of cytosolic iron-sulfur cluster assembly and delivery.

## Methods

### ScMet18 mutagenesis and purification

The cloning of ScMet18 with an N-terminal His-tag followed by SUMO solubility tag (^SUMO^Met18) was created by amplifying the *Met18* gene from genomic DNA using primers AV03 and AV04^13^. *Met18* was inserted into a pRSF-Duet vector in between *Eco*RI and *Sal*I sites via Gibson DNA assembly^13^. A SUMO coding sequence was inserted at the *Eco*RI using primers AV26 and AV27^13^. All mutations were introduced by Q5 mutagenesis kit (New England Biolabs) according to manufacturer’s instructions and confirmed by DNA sequencing (Genewiz).

The plasmid for expression of wild-type ^SUMO^ScMet18 or the desired variant was transformed into Rosetta2(DE3), and protein expression was induced by autoinduction at room temperature. Autoinduction media (1 L) contained 6 g Na_2_HPO_4_, 3 g KH_2_PO_4_, 5 g NaCl, 10 mL 60% glycerol, 5 mL 10% glucose, 25 mL 8% lactose. For purification, cell paste was resuspended in Buffer A (50 mM Tris-HCl (pH 8), 100 mM NaCl, 5% glycerol and 5 mM β-mercaptoethanol (BME)) and lysed by sonication. Met18 was purified from the soluble extract using Ni-NTA resin, and it was exchanged into buffer B (50 mM Tris-HCl (pH 8), 100 mM NaCl, 1% glycerol, 1 mM BME) via gel filtration chromatography, and concentrated to 2.5 mg/mL (19 µM) for mass photometry studies^13^.

For removal of His-SUMO tag, His-tagged SUMO protease (2 mg or 0.074 µM) was added to ^SUMO^ScMet18 (25 mg or 0.191 µM) and incubated overnight at 4˚C. The mixture was passed over a Ni-NTA column and untagged ScMet18 was recovered from the flow through and concentrated. For cryo-EM analysis, samples were freshly prepared, avoiding freeze-thaw cycles, and diluted to the desired concentration in buffer C (50 mM HEPES, 100 mM NaCl, 1% glycerol, 1 mM BME).

### Pulldown assays

For protein-protein interaction analysis, pulldown assays using double tagged ScCia2 (^DT^Cia2, with N-terminal strep tag and C-terminal His tag), ScCia1 (^His^Cia1 with N-terminal His tag), ^SUMO^ScMet18 and ScLeu1 (^His^Leu1 with N-terminal His tag) were performed as described^12,13^. Briefly, the strep-tagged ScCia2 bait (~2 µM) was mixed in equimolar amounts with the indicated prey proteins (^SUMO^Met18, ^His^Cia1, and/or ^His^Leu1, as indicated) for 1 h at 4˚C in Buffer A. The mixture was batch absorbed to Strep-Tactin XT superflow resin (IBA), and then resin was collected, washed, and eluted according to manufacturer instructions. Fractions containing the highest concentration of protein were analyzed by SDS-PAGE in parallel with samples derived from Strep-Tactin input. In all cases, a negative control, in which the ^DT^Cia2 bait was omitted, and a positive control, utilizing wild-type ScMet18, were completed in parallel.

### Mass photometry

Data were collected using AcquireMP (Refeyn Ltd) and analyzed using DiscoverMP (Refeyn Ltd). Each Mass Photometry (MP) measurement was performed for 60 sec. Samples were diluted in 50 mM Tris pH 8.0, 100 mM NaCl, 1% glycerol, and 5 mM BME to final concentrations of 0.050 µM ^SUMO^ScMet18. For SUMO-cleaved ScMet18, MP was conducted at 0.050 µM. Untagged ScMet18 in the following complexes: ScMet18-ScCia2, ScMet18-ScCia1, and ScMet18-ScLeu1 were all performed at 0.050 µM, as well. The molecular weights were obtained using contrast comparison with known molecular weights from mass standard calibrants measured the same day.

### Vitrobot cryo-EM specimen preparation

The sample was prepared using a Quantifoil R 1.2/1.3 Au 300 mesh holey-carbon grid and plunged using a Thermo Fisher Scientific Vitrobot cryo-plunger. The grid was glow discharged at −15 mA for 1 min (PELCO easiGlow) before protein solution was applied. ScMet18 (untagged, His-SUMO tag removed by SUMO protease treatment) was diluted to a final concentration of 1.2 mg/mL (9 µM) in Buffer C (50 mM HEPES pH 7.5, 100 mM NaCl, 1% glycerol, and 1 mM BME) and 33 mM sodium acetate was added to the solution. The use of this buffer composition and this protein concentration was empirical; it allowed for good distribution of ScMet18 particles on grids, limiting the crowding of particles. ScMet18 (3 μL) was applied to the grid and was blotted for 6 sec before plunging into liquid ethane and transferring to storage buttons. The temperature and humidity were 10 °C and 95%, respectively.

### Cryo-EM data collection for the 0°, 25°, and 40° tilted datasets

Data were collected at the University of Massachusetts Medical School at Worcester Cryo-EM Core Facility on a Thermo Fisher Titan Krios 300 kV electron microscope equipped with a Gatan GIF K2 camera at 130000x magnification across three collections. All three data collections were from the same protein preparation. Parameters for the 0° tilted dataset were as follows: 1.059 Å/pix (collected at super-resolution of 0.529 Å /pix), 45 frames, 1.102 electrons/ Å^2^ /frame dose, −0.7– −2.5 µm defocus range. This dataset contained 3968 frames. Parameters for the 25° and 40° tilted dataset were as follows: 1.059 Å /pix (collected at super-resolution of 0.529 Å /pix), 45 frames, 1.104 electrons/ Å^2^/frame dose, defocus range −0.8– −2.4 µm. The 25° tilted dataset contained 1783 frames, and the 40° tilted dataset contained 2280 frames (Table 1).

### Frame alignment, defocus estimation, and micrograph assessment in SPHIRE of the 0°, 25°, and 40° tilted datasets

Each individual frame of dose-fractionated exposure was summed and aligned using MotionCor2^26^. For the 0° tilted datasets, the defocus of the summed frames was estimated using CTER^27^. For the 25° and 40° tilted datasets, the defocus of the summed frames was estimated using GCTF^28^. Outputs from motion_cor2 and CTER were used to perform drift and CTF assessments within the SPHIRE software suite^29^. The final number of micrographs for the 0°, 25°, and 40° tilted datasets were 3612, 1646, and 2079, respectively, for a total of 7337 micrographs used for selection moving forward (Table 1).

### Particle selection of the 0°, 25°, and 40° tilted datasets

For the 0-degree tilted dataset, ~1800 particles were picked manually from a subset of aligned movies. The neural-net automated particle picker Topaz^30^ was used to automatically pick particles from the entire dataset. Using a cutoff threshold of −2, the initial particle hits resulted in 172848 particles. For the 25-degree tilted dataset, ~1200 particles were picked manually from a subset of aligned movies. Topaz^30^ was used to automatically pick particles from the entire dataset. Using a cutoff threshold of −2, the initial particle hits resulted in 136153 particles. For the 40-degree tilted dataset, ~1600 particles were picked manually from a subset of aligned movies. Topaz^30^ was used to automatically pick particles from the entire dataset. Using a cutoff threshold of −3, the initial particle hits resulted in 114057 particles.

### Frame alignments, map generations, and refinement in Relion 3.0 of the 0°, 25°, and 40° tilted datasets

Frame alignment using motion_cor2 was rerun on all three datasets within the Relion 3.0 software suite^31^. CTF estimation of the 0-degree tilt dataset was also rerun using CTFFIND4^32^ on the output from Relion MotionCor2. A total of 418832 particles from Topaz were used to re-extract particles (box-size 330 pix) and perform initial reference-free 2D classification (mask diameter 200 Å for 25- and 40-degree tilt datasets, 190 Å for 0-degree tilted dataset). Reference-free 2D classification was performed separately for each dataset and 379779 particles were pooled to generate the ab initio reference-free 3D model applying no symmetry (C1) and D3 symmetry (Fig. S2). The D3 map was then subjected to 3D classification assigning two classes. Class 1 which had 171255 particles was then subjected to 3D-refinement with an initial low-pass filter of 50 Å. CTF refinement and Bayesian polishing were performed on this map, and another round of 3D-refinement and post processing was performed, resulting in a 3.33 Å resolution map (Map 1). The mask applied during post-processing was low-pass filtered by 15 Å with an initial binarization threshold of 0.006 extended by 5 pixels with a soft edge of 6 pixels.

The map was subjected to focused classification to resolve the 3-fold N-terminal density (Fig. S1). Focused masks were made in Chimera^33^ and Relion 3.0^31^ and low-pass filtered by 15 Å with an initial binarization threshold of 0.004 extended by 5 pixels with a soft edge of 6 pixels. Using this mask, 3D classification was performed to classify the map into three classes. Class 3 of 44707 particles was subjected to reference-free masked 3D refinement and post-processing to obtain a final map of 3.57 Å resolution (Map 2). Maps 1 and 2 were combined using WARP Frankenmap^17^, and this combined map was used for model building and refinement.

### Model building and refinement of the ScMet18 hexamer structure from the 0°, 25°, and 40° tilted datasets

Residues 138–1029 were manually built into the combined map using Coot^34^. The MmMet18 structure (PDB 6TC0) was docked into the map using Coot and used as a starting model for building residues 9-137. Multiple rounds of refinement were carried out in Phenix^35^ with model building in Coot^34^. The final ScMet18 hexamer model contains residues 9-225, 242–314, and 338–1029. Figures of the map and model were made using UCSF ChimeraX^36^. Coot, Phenix, and ChimeraX were licensed through the SBGrid Consortium operated out of Harvard Medical School^37^. Refinement and model statistics are summarized in Tables 1 and 2.

### Chameleon cryo-EM specimen preparation and data collection

A specimen was prepared on a chameleon grid with 1.2 μm holes with 0.8 μm spacing. The grid was glow discharged at −12 mA for 210 s (PELCO easiGlow) before protein solution was applied. The concentration of untagged ScMet18 was at 2 μg/μL (15 µM) in 50 mM Tris pH 8.0, 100 mM NaCl, 1% glycerol, and 1 mM BME. After ScMet18 was applied to the grid, the grid self-wicked for 569 ms before plunging into liquid ethane and transferring to storage buttons. The temperature and humidity were 24 °C and 77%, respectively.

Data were collected at the MIT.nano Cryo-EM Facility on a Talos Arctica 200 kV electron microscope equipped with a Falcon3 camera at 92000x magnification. Parameters for the dataset were as follows: 1.5998 Å/pix, 14 frames, 3.804 electrons/ Å^2^ /frame dose, −1.3 - −3.4 µm defocus range. This dataset contained 483 frames (Table 1).

### Chameleon cryo-EM data processing

Data were processed in cryoSPARC v3.2.0^38^. Frame alignment was performed using Patch motion correction. CTF estimation was estimated using the CTFFIND4 wrapper^32^. Roughly 1000 particles were manually picked using the Manual Picker and these particles were subjected to 2D classification to obtain template for automatic picking using the Template Picker. The Template Picker picked 460492 number of particles, and these particles inspected and a final stack of 139569 number of particles were used for 2D classification. 87417 particles were used as inputs for 3D ab initio modeling into 3 models. Models 1 (30104 particles) and 2 (36582 particles) were subjected to one round of non-uniform Refinement to obtain final maps 4 and 3, respectively. Map 3 is at a final resolution of 8.46 Å, and map 4 is at a final resolution of 12.77 Å (Figs. S7 and S8).

### Model building and refinement of the ScMet18 tetramer structure

Chains A, C, D, and F (each containing residues 9-225, 242-314, and 338-1029) from the 3.3 Å resolution structure of the ScMet18 hexamer were docked into the 12.8 Å resolution map (map 4) using Phenix Dock In Map^35^. One round of rigid-body real space refinement was carried out in Phenix^35^. Figures of the map and model were made using UCSF ChimeraX^36^. Coot, Phenix, and ChimeraX were licensed through the SBGrid Consortium operated out of Harvard Medical School^37^. Refinement and model statistics are summarized in Tables 1 and 2.

### Reporting summary

Further information on research design is available in the Nature Portfolio Reporting Summary linked to this article.

### Supplementary information

Supplementary Information

Description of Additional Supplementary Data

Supplementary Movie

Reporting Summary

## Data Availability

The cryo-EM density maps of the *S. cerevisiae* Met18 hexamer from Vitrobot sample preparation method, Map 1, Map 2, and the composite map, have been deposited to the Electron Microscopy Data Bank (EMDB) under accession numbers EMD-42512, and EMD-42513, and EMDB-42510, respectively. The *S. cerevisiae* Met18 hexamer Map 3 from the chameleon sample preparation method has been deposited to the EMDB under accession number EMD-42514. The *S. cerevisiae* Met18 tetramer Map 4 from the chameleon sample preparation has been deposited to the EMDB under accession number EMD-42511. The *S. cerevisiae* hexamer and tetramer cryo-EM structures have been deposited to the Protein Data Bank (PDB) under accession numbers 8USP and 8USQ, respectively. Unedited gels from the CTC pulldown assays are found in Supplementary Fig. 10. All relevant data are available from the authors upon request.
