# Supplementary material for: Structural and biochemical investigations of a HEAT-repeat protein involved in the cytosolic iron-sulfur cluster assembly pathway
Source: Commun Biol. 2023 Dec 18;6:1276. doi: 10.1038/s42003-023-05579-3 (PMC10728100; doi:10.1038/s42003-023-05579-3)
Supplement: Supplementary file 1 — Supplementary Information [file 42003_2023_5579_MOESM1_ESM.pdf]

Structural and biochemical investigations of a HEAT-repeat protein involved in the cytosolic iron-sulfur cluster assembly pathway

Supplementary Information

Sheena Vasquez[1], Melissa D. Marquez[2], Edward J. Brignole[1,3], Amanda Vo[2], Sunnie Kong[2], Christopher Park[2], Deborah L. Perlstein[2*], and Catherine L. Drennan[1,4,5*]

[1]Department of Biology, Massachusetts Institute of Technology, Cambridge, MA 02139 USA
[2]Department of Chemistry, Boston University, Boston, MA 02215 USA
[3]MIT.nano, Massachusetts Institute of Technology, Cambridge, MA 02139 USA
[4]Howard Hughes Medical Institute, Massachusetts Institute of Technology, Cambridge, MA 02139 USA
[5]Department of Chemistry, Massachusetts Institute of Technology, Cambridge, MA 02139 USA

Content:
Supplemental Figures S1-S10

*To whom correspondence should be addressed: cdrennan@mit.edu; dperl@bu.edu

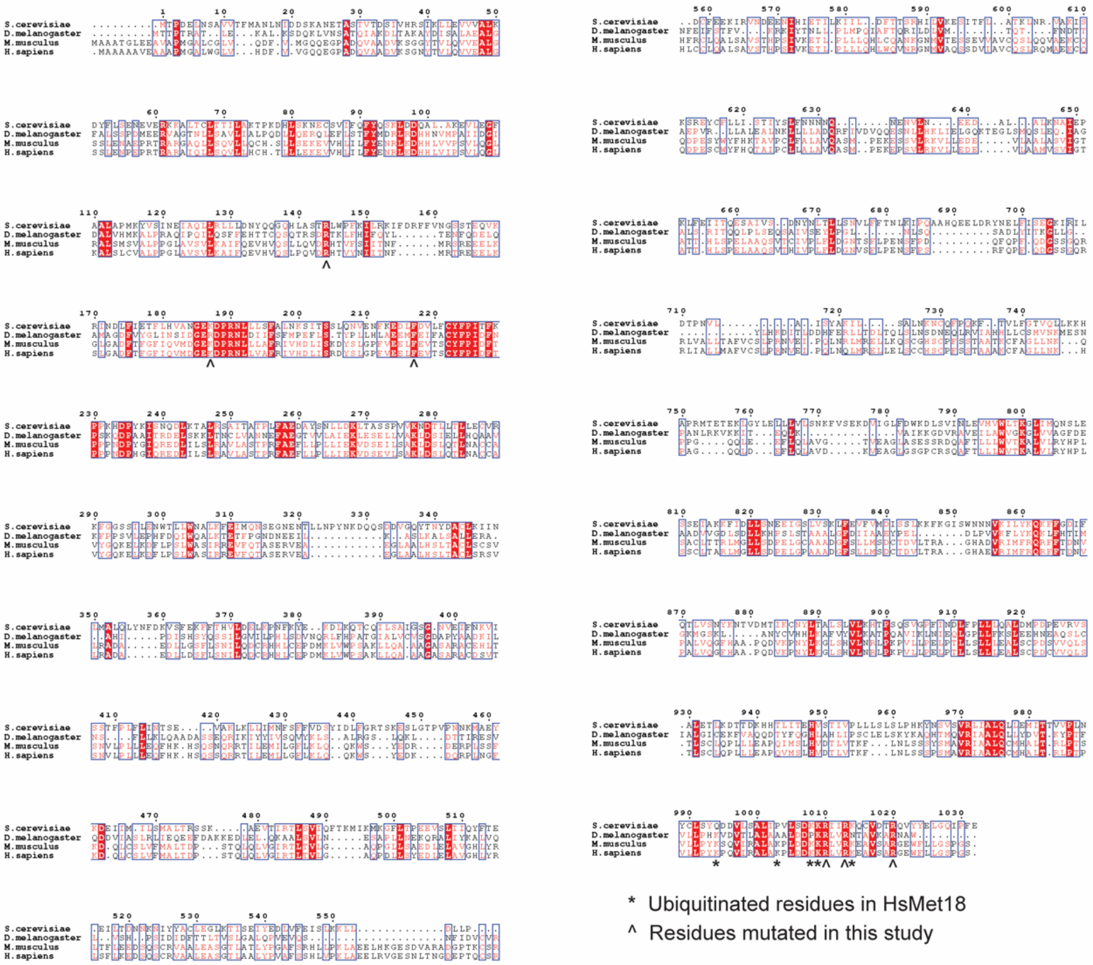

Figure S1. Sequence alignment of Met18. Secondary structure features are depicted for ScMet18 and MmMet18. Highlighted in red are conserved residues. Residues mutated in this study are indicated by ^: residues R144, K187, and F217 at the N-terminus and R1010, R1013, and R1020 at the C-terminus. K1008, K1009, and K1014 are indicated by * along with conserved residues targeted to the proteosome for degradation. Sequence alignment was made using Clustal Omega (https://www.ebi.ac.uk/Tools/msa/clustalo/) and figure was made using ESPript 3 (https://espript.ibcp.fr/ESPript/ESPript/).

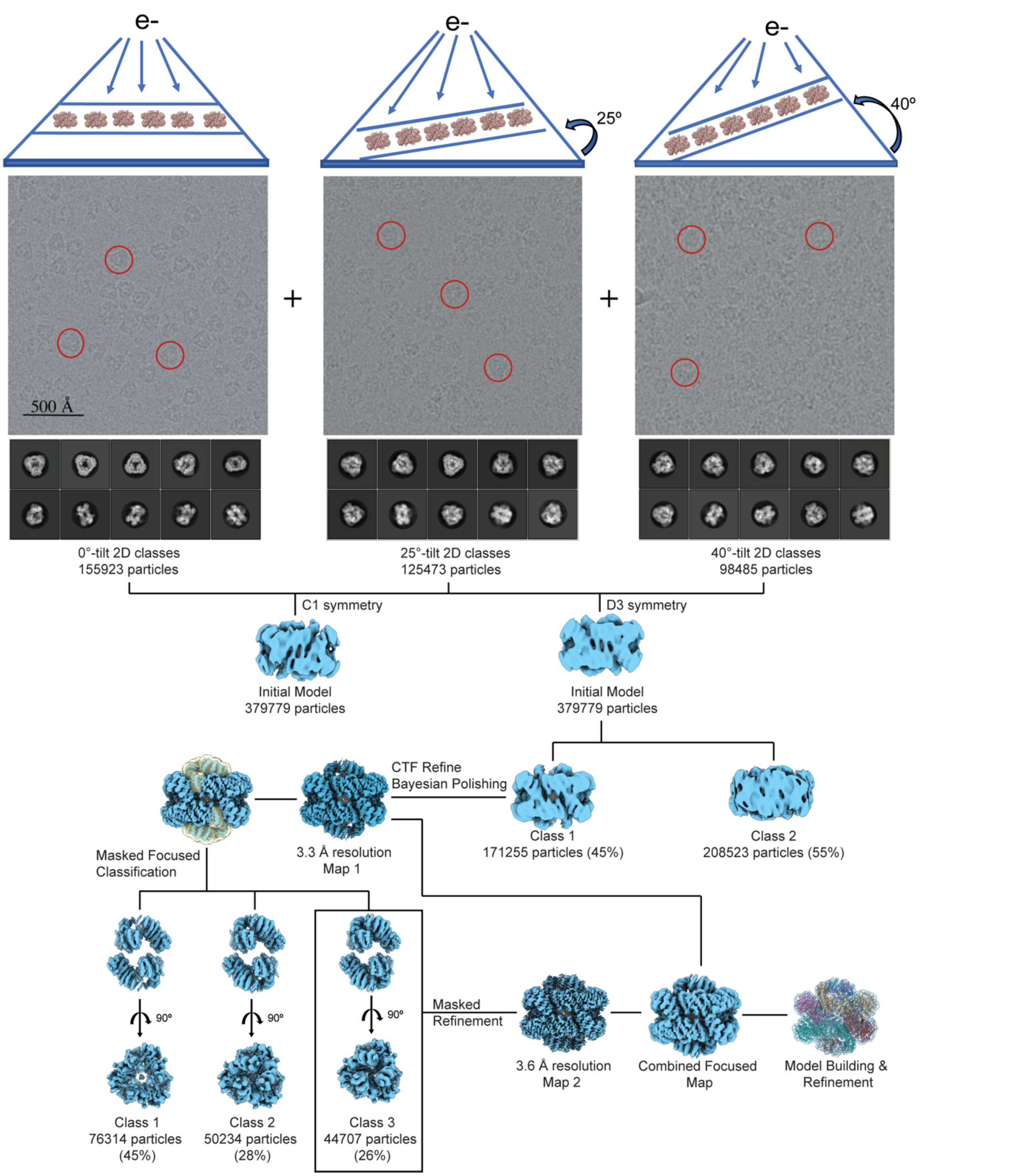

Figure S2. Workflow of Vitrobot-prepared cryoEM data processed to obtain a 3.3 Å resolution structure of the ScMet18 hexamer. Three datasets were collected applying 0°, 25°, and 40° stage tilts. Representative particles are circled in red. Particles were picked using Topaz and the datasets were processed individually up until 2D classification in Relion, and then the datasets were pooled to create the 3D initial models. Two initial models were made applying no symmetry (C1) and D3 symmetry consisting of 379779 particles, each. Because the C1 map displayed symmetry, D3 symmetry was applied throughout the data processing. After 3D classification, the Class 1 map, which consisted of 171255 particles, was further refined to 3.3 Å resolution (Map 1). Map 1 was then subjected to masked focused classification, and particles from Class 3 (boxed above) were subjected to a masked refinement to generate a 3.6 Å resolution map (Map 2). The choice of particles from Class 3 was empirical; the map generated (Map 2) from Class 3 particles had better density for the ScMet18 N-terminus. Maps 1 and 2 were combined into a combined focused map using Frakenmap and used for model building and refinement [17].

a.

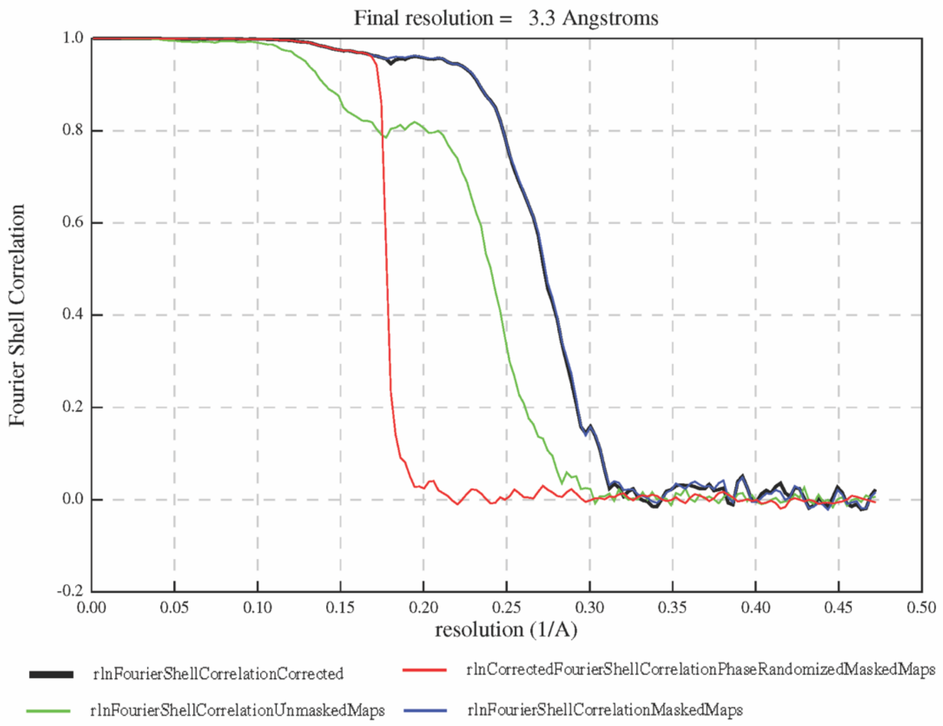

b.

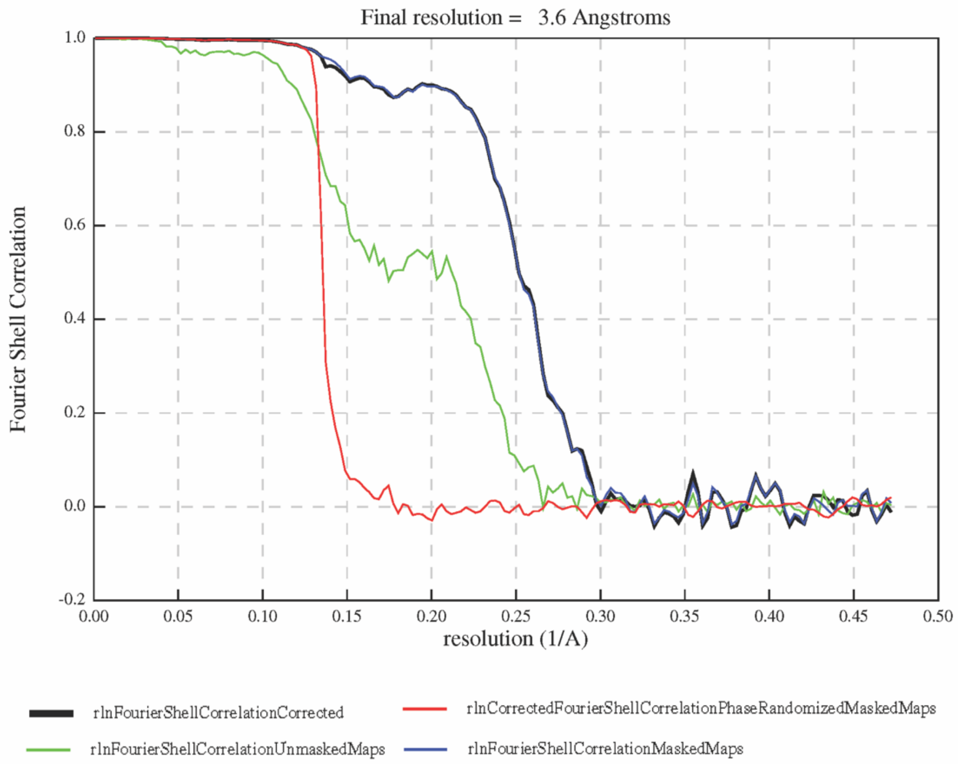

Figure S3. FSC plots for maps of ScMet18 hexamer generated using Vitrobot-prepared particles. A. FSC plots for masked (blue) and unmasked (green) Map 1. For the masked map, the resolution is at 3.3 Å at a 0.143 cutoff. **B.** FSC plots for masked (blue) and unmasked (green) Map 2. For the masked map, the resolution is at 3.6 Å at a 0.143 cutoff. Both maps contain data from the tilted and untilted datasets. The difference between maps is shown in Figure S2.

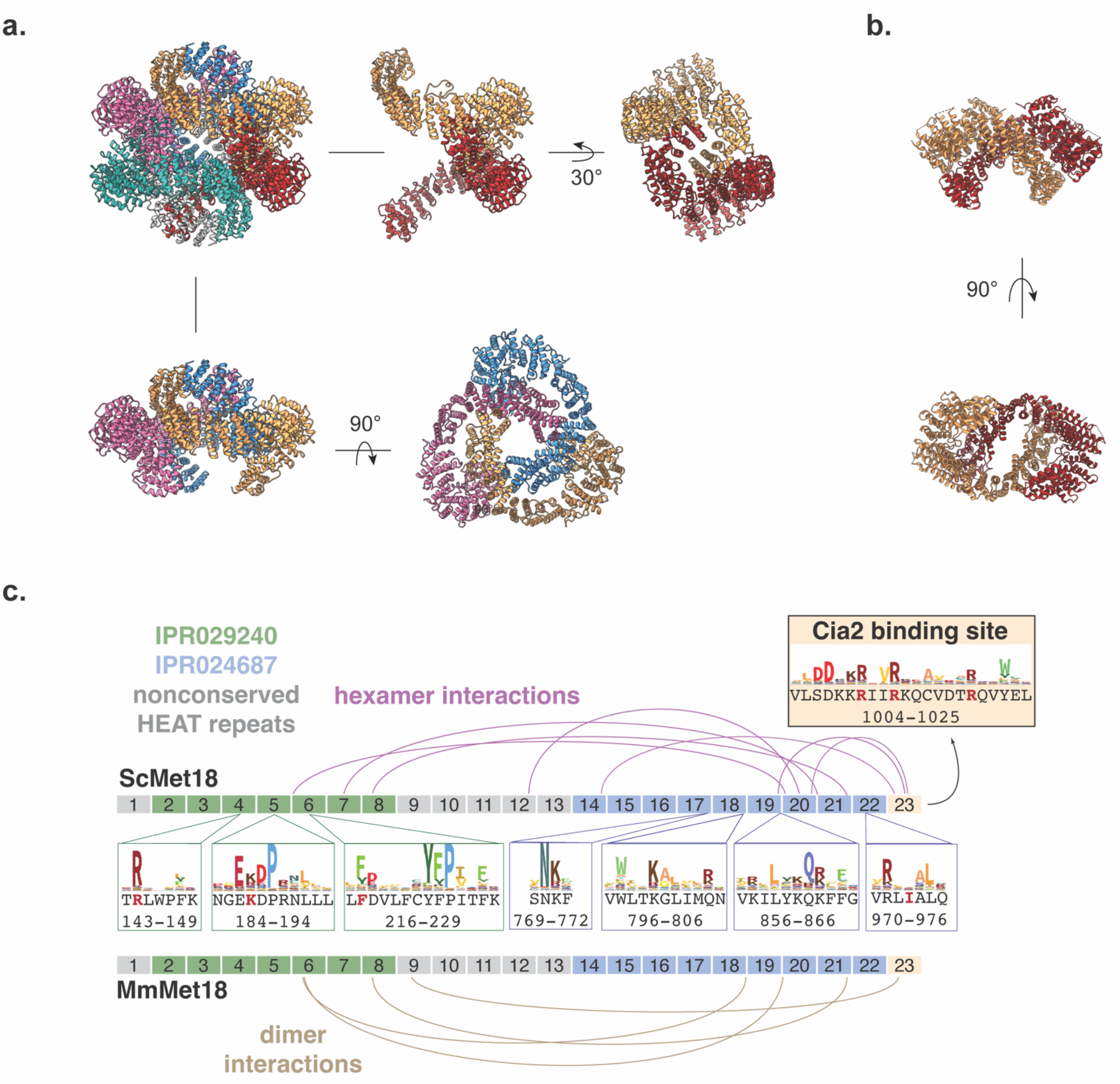

Figure S4. The ScMet18 hexamer is composed of dimers and trimers. A. The hexamer (top left) is composed of two trimers (bottom) with chains A (sandy brown), B (dodger blue), and C (hot pink) interacting (trimer shown), and D (cyan), E (grey) and F(red) interacting (trimer not shown). The hexamer is also made up of three dimers with chains A (sandy brown) and F (red) interacting (dimer on top right), chains B (dodger blue) and E (grey) (not shown), and chains C (hot pink) and D (cyan) interacting (not shown). Each chain interacts with four other chains. For example, chain A (sandy brown) interacts with chain B (dodger blue), chain C (hot pink), chain D(cyan), and chain F (red), but not chain E (grey). **B.** The MmMet18 dimer from the crystal structure of MmMet18 in complex with DmCia2b-DmCia1 is not the same as the dimer observed within the ScMet18 hexamer. The chains of the MmMet18 dimer are colored in sandy brown and red for comparison with the dimer in ScMet18. **C.** ScMet18 and MmMet18 have the same number of HEAT-like repeats. Purple and orange lines depict hydrogen bonding networks. Most of the interactions are made from highly conserved HEAT-repeats (green and blue). Insets are weblogos from aligning the InterPro website aligning 100 Met18 sequences. Red letters in sequences under the WebLogos (https://weblogo.berkeley.edu/logo.cgi) depict residues substituted in this study. The Cia2 weblogo was made from aligning 70 Met18 sequences.

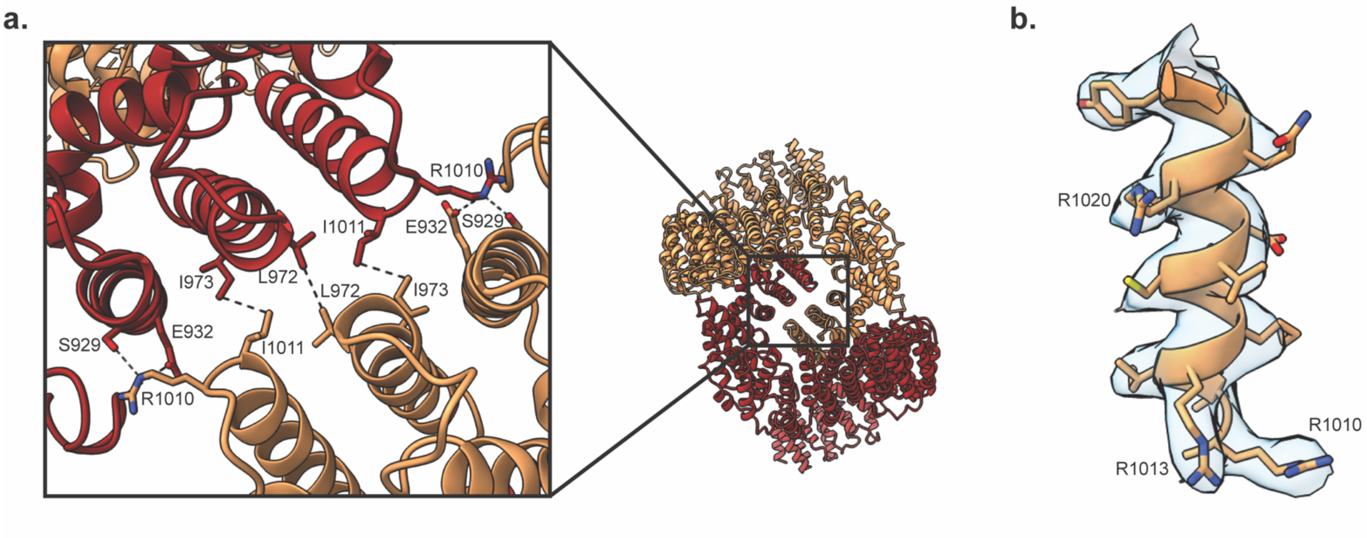

Figure S5. Hexameric structure of Met18 is stabilized by both hydrophobic packing and polar interactions. **A.** Interactions of at the dimer interface of the A (shady brown) and F (red). **B**. Representative side chain density with C-terminal residues labeled that were substituted in this study.

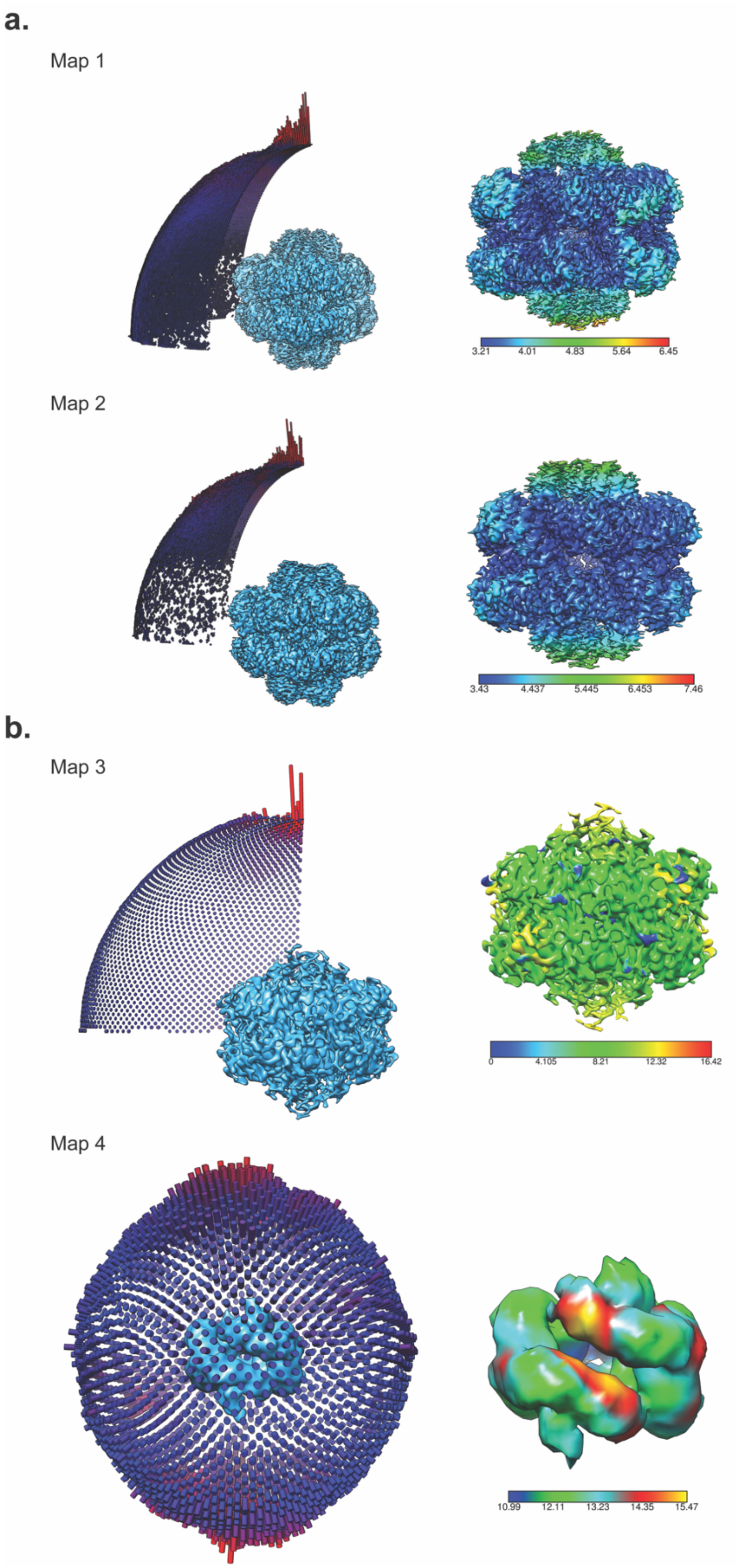

Figure S6. Angular distribution Plots and Local Resolution Graphs. A. Vitrobot-prepared sample collected on Krios using stage tilt. The angular distribution plots for Maps 1 and 2 show high preferred orientation at the top 3-fold axis of ScMet18. The resolution for Map 1 ranges between 3.21 – 6.45 Å, and for Map 2 the resolution ranges between 3.43 – 7.46 Å. Both maps contain data from the tilted and untilted datasets. The difference between maps is shown in Figure S2. **B.** chameleon-prepared sample collected on Arctica without stage tilt. The angular distribution plot for Map 3 shows high preferred orientation at the top 3-fold axis of ScMet18. The resolution for Map 3 ranges between 0 – 16.42 Å. The angular distribution plot for Map 4 shows uniform distribution with particles contributing to the most views at the top and bottom views. For Map 4, the resolution ranges between 10.99 – 15.47 Å.

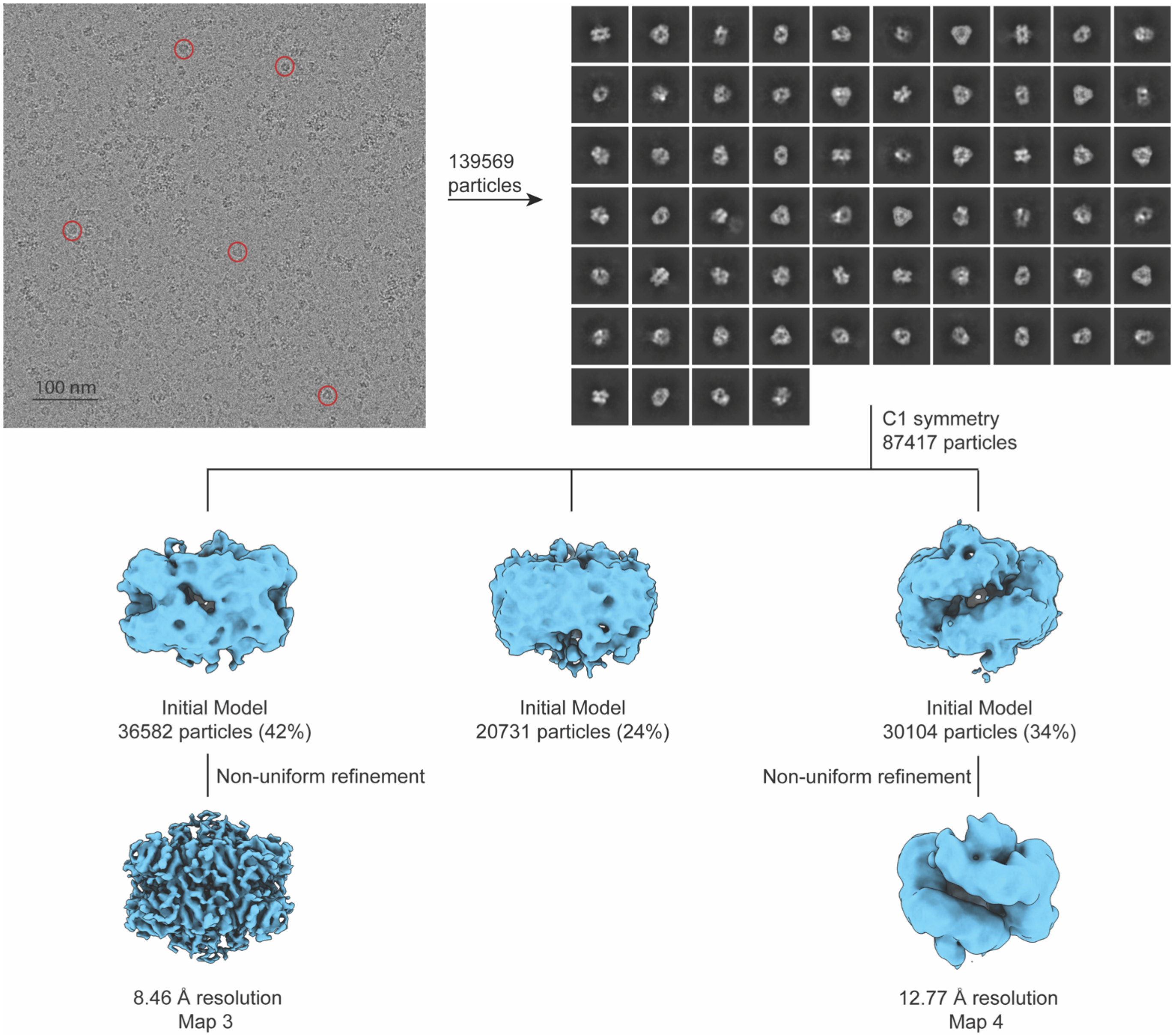

Figure S7. Workflow of chameleon-prepared cryo-EM data processed to obtain an addition map of ScMet18 hexamer and a map for a ScMet18 tetramer structure. One small dataset was collected and processed in cryoSPARC [32]. Representative particles are circled in red. 139569 particles were classified in 2D space and 87417 of those particles were further used for *ab initio* modeling. Three models were made applying C1 symmetry. Map 3 was generated with 42% of the particles after applying D3 symmetry and performing non-uniform refinement. Map 3 refines to 8.46-Å resolution. Map 4 was generated with 34% of the particles after applying C1 symmetry and performing non-uniform refinement. Map 4 refines to 12.77 Å resolution.

a.

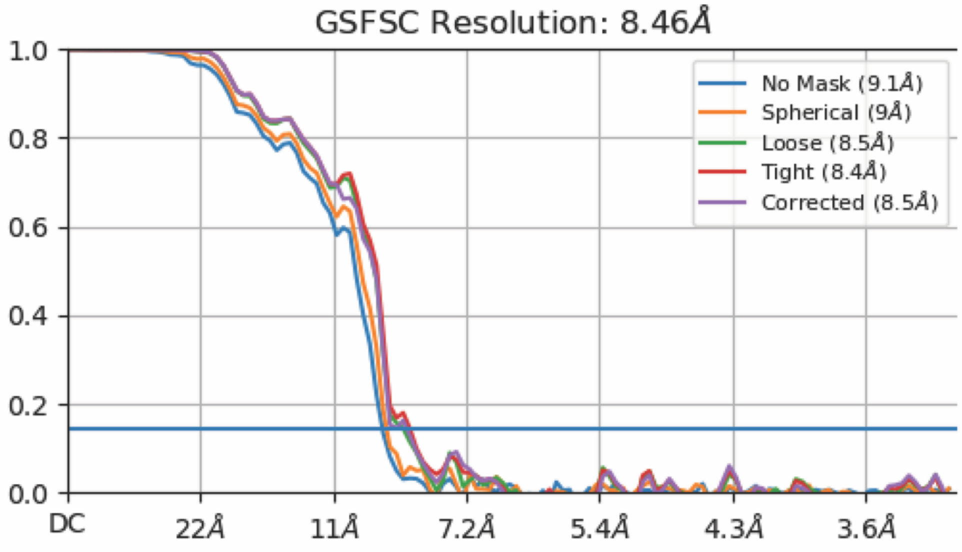

b.

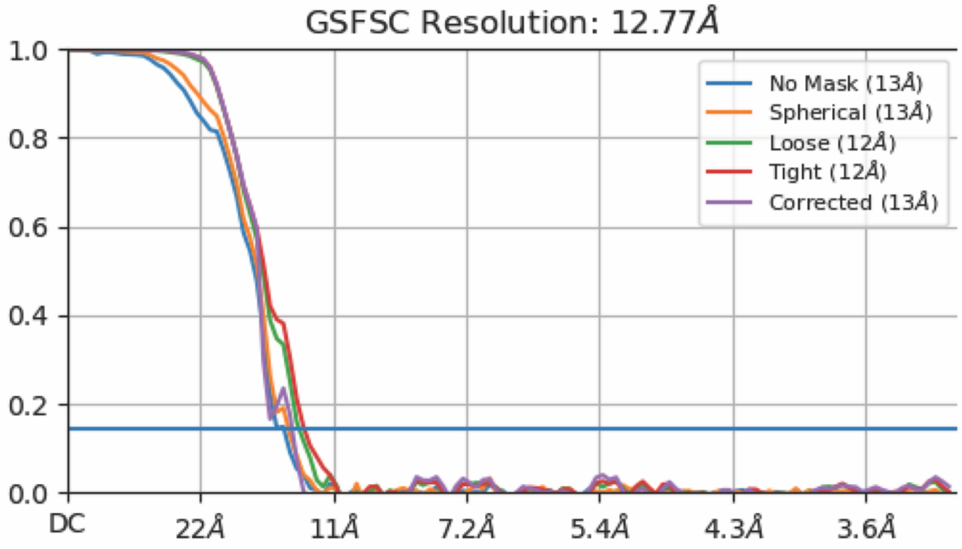

Figure S8. FSC plots for maps of ScMet18 hexamer and tetramer generated using chameleon-prepared particles. A. FSC plot for masked (red) and unmasked (blue) map 3 (hexamer). For the masked map, the resolution is at 8.4 Å at a 0.143 cutoff. **B**. FSC plots for masked (red) and unmasked (blue) map 4 (tetramer). For the masked map, the resolution is at 12 Å at a 0.143 cutoff.

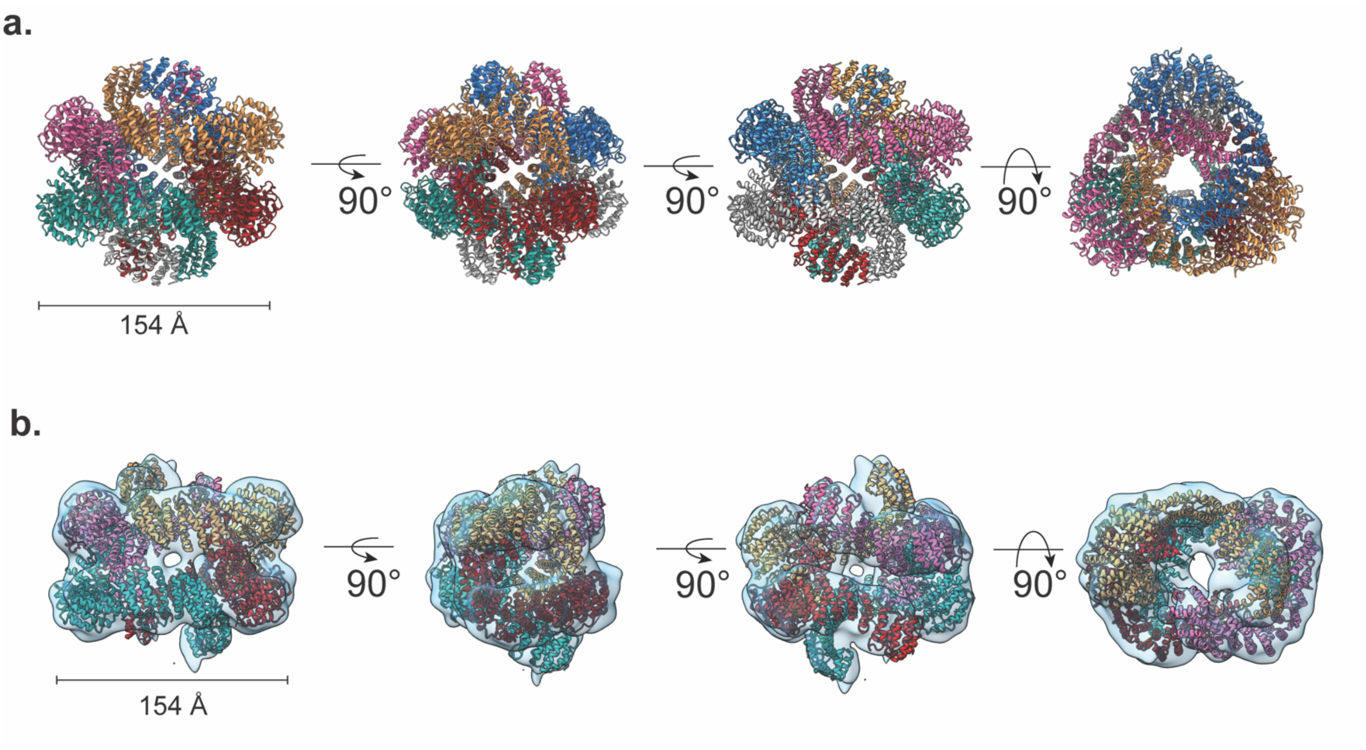

Figure S9. ScMet18 can form tetramers and hexamers. A. ScMet18 hexamer with each chain colored a different color for clarity. The ScMet18 hexamer has 3-fold and 2-fold symmetry and is 154 Å. **B.** ScMet18 tetramer with each chain colored a different color for clarity. The ScMet18 tetramer has 2-fold symmetry and is 154 Å in length. Similar orientations of the ScMet18 hexamer and tetramer are aligned.

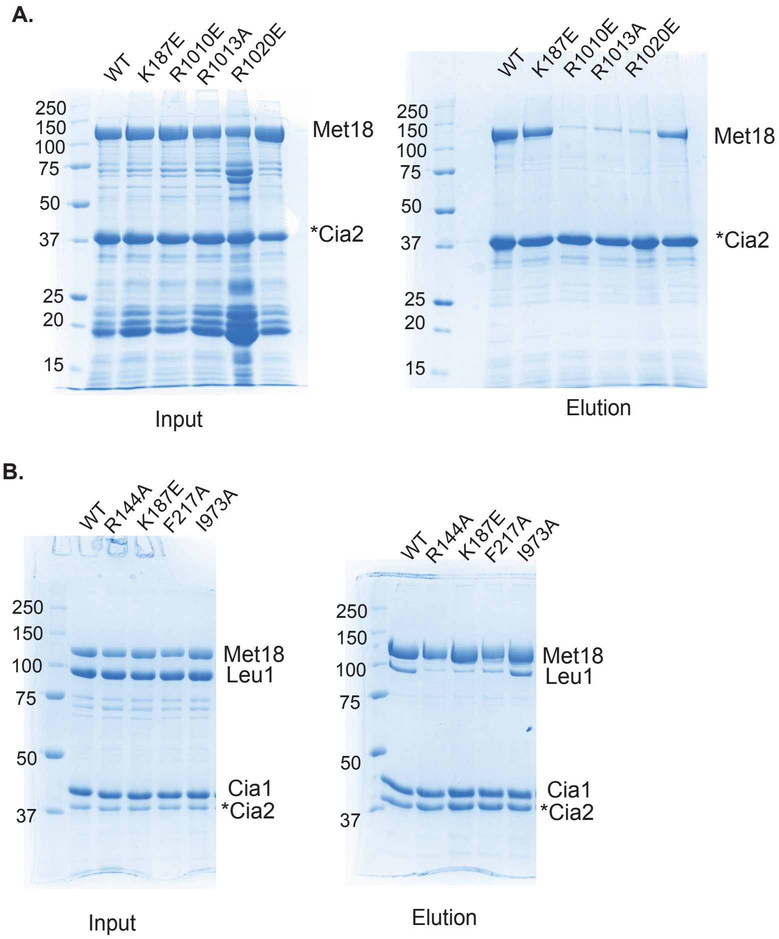

Figure S10. Unedited SDS-PAGE images of Figure 3. A. Unedited SDS-PAGE image of Figure 3B. **B**. Unedited SDS-PAGE image of Figure 3C.

17. Farnung, L., M. Ochmann, M. Engeholm, and P. Cramer, *Structural basis of nucleosome transcription mediated by Chd1 and FACT.* Nat Struct Mol Biol, 2021. **28**(4): p. 382-387.
