# Supplementary material for: Structural and biochemical investigations of a HEAT-repeat protein involved in the cytosolic iron-sulfur cluster assembly pathway
Source: Commun Biol. 2023 Dec 18;6:1276. doi: 10.1038/s42003-023-05579-3 (PMC10728100; doi:10.1038/s42003-023-05579-3)
Supplement: Supplementary file 2 — Description of Additional Supplementary Data [file 42003_2023_5579_MOESM2_ESM.docx]

**Description of Additional Supplementary Files**

**File name:** Supplementary Movie 1

**Description:** The Met18 hexamer combined focus map with the Met18 hexamer model.
